# Microbiomic differences in tumor and paired-normal tissue in head and neck squamous cell carcinomas

**DOI:** 10.1186/s13073-017-0405-5

**Published:** 2017-02-07

**Authors:** Hannah Wang, Pauline Funchain, Gurkan Bebek, Jessica Altemus, Huan Zhang, Farshad Niazi, Charissa Peterson, Walter T. Lee, Brian B. Burkey, Charis Eng

**Affiliations:** 1 Genomic Medicine Institute, Lerner Research Institute, Cleveland, OH 44195 USA; 2 Cleveland Clinic Lerner College of Medicine, Cleveland, OH 44195 USA; 3 Taussig Cancer Institute, Cleveland, OH 44195 USA; 4 Head and Neck Institute, Cleveland Clinic, Cleveland, OH 44195 USA; 5 Department of Surgery, Duke University Medical Center, Durham, NC 27710 USA; 6 Center for Proteomics and Bioinformatics, Cleveland, OH 44106 USA; 7 Department of Electrical Engineering and Computer Science, Cleveland, OH 44106 USA; 8 Department of Genetics and Genome Sciences, Cleveland, OH 44106 USA; 9 CASE Comprehensive Cancer Center, Case Western Reserve University School of Medicine, Cleveland, OH 44106 USA; 10 Cleveland Clinic Genomic Medicine Institute, 9500 Euclid Avenue NE50, Cleveland, OH 44195 USA

**Keywords:** Head and neck squamous cell carcinoma (HNSCC), Bacteria, Microbiome

## Abstract

**Background:**

While the role of the gut microbiome in inflammation and colorectal cancers has received much recent attention, there are few data to support an association between the oral microbiome and head and neck squamous cell carcinomas. Prior investigations have been limited to comparisons of microbiota obtained from surface swabs of the oral cavity. This study aims to identify microbiomic differences in paired tumor and non-tumor tissue samples in a large group of 121 patients with head and neck squamous cell carcinomas and correlate these differences with clinical-pathologic features.

**Methods:**

Total DNA was extracted from paired normal and tumor resection specimens from 169 patients; 242 samples from 121 patients were included in the final analysis. Microbiomic content of each sample was determined using 16S rDNA amplicon sequencing. Bioinformatic analysis was performed using QIIME algorithms. F-testing on cluster strength, Wilcoxon signed-rank testing on differential relative abundances of paired tumor-normal samples, and Wilcoxon rank-sum testing on the association of T-stage with relative abundances were conducted in R.

**Results:**

We observed no significant difference in measures of alpha diversity between tumor and normal tissue (Shannon index: *p* = 0.13, phylogenetic diversity: *p* = 0.42). Similarly, although we observed statistically significantly differences in both weighted (*p* = 0.01) and unweighted (*p* = 0.04) Unifrac distances between tissue types, the tumor/normal grouping explained only a small proportion of the overall variation in the samples (weighted R^2^ = 0.01, unweighted R^2^ < 0.01).

Notably, however, when comparing the relative abundances of individual taxa between matched pairs of tumor and normal tissue, we observed that *Actinomyces* and its parent taxa up to the phylum level were significantly depleted in tumor relative to normal tissue (*q* < 0.01), while *Parvimonas* was increased in tumor relative to normal tissue (*q* = 0.01). These differences were more pronounced among patients with more extensive disease as measured by higher T-stage.

**Conclusions:**

Matched pairs analysis of individual tumor-normal pairs revealed significant differences in relative abundance of specific taxa, namely in the genus *Actinomyces*. These differences were more pronounced among patients with higher T-stage. Our observations suggest further experiments to interrogate potential novel mechanisms relevant to carcinogenesis associated with alterations of the oral microbiome that may have consequences for the human host.

**Electronic supplementary material:**

The online version of this article (doi:10.1186/s13073-017-0405-5) contains supplementary material, which is available to authorized users.

## Background

Interactions between microbes and carcinogenesis within the host have been studied for decades. The best example is in the context of a single microorganism: *Helicobacter pylori* and its proven link with gastric cancer [1]. At the other end of the spectrum, and more recently, *Fusobacterium nucleatum* was described in the gut of those with advanced colorectal cancer [2, 3]. Subsequent functional studies demonstrated *F. nucleatum* to be capable of both upregulating inflammatory and oncogenic pathways in colon cancer cell lines [4] and inducing adenomas in mice [5]. The bacterial microbiome, defined as the total collection of bacteria that inhabit any environmental niche, has been increasingly recognized as an active participant in human body functions and proposed to be an organ in its own right. At a basic level, we have long understood that the microbiome serves to maintain homeostasis. Dysbiosis, or disruption of the normal flora, can result in pathogenic overgrowth of organisms including *Clostridium* and *Candida* in the gastrointestinal and vaginal tracts, respectively [6–8]. Similarly, the oral microbiome has long been studied in the context of dental caries: ingestion of excessive carbohydrates promotes overgrowth of acidogenic and acidophilic microbes, inducing a local drop in pH, demineralization of enamel, and subsequent breakdown of tooth [9, 10].

While investigation of the association between microbial dysbiosis and colorectal cancer is progressing at a rapid pace, the study of the bacterial microbiome in other areas of the gastrointestinal tract has lagged behind. In particular, head and neck squamous cell carcinomas (HNSCC), which account for more than half a million cancer cases annually around the world [11, 12], has received relatively little attention. This may be due to the fact that HNSCC is a heterogeneous disease entity, encompassing a variety of cancers from different disease sites, and develops from the mucosal linings of the upper aerodigestive tract, comprising: (1) the nasal cavity and paranasal sinuses; (2) the nasopharynx; (3) the oral cavity and oropharynx; and (4) the hypopharynx, larynx, and trachea [13]. Additionally, major risk factors such as smoking, alcohol consumption, and human papillomavirus infection have already been elucidated [13, 14].

However, recent studies have identified microbiomic shifts in the oral cavity associated with cigarette smoking, and in the gastrointestinal tract associated with alcohol consumption [15, 16]. Similar to the pathogenesis of dental caries, it is feasible that the microbiome helps transduce an environmental exposure into a carcinogenic effect. As there are few effective systemic therapies in HNSCC, and toxicity of local treatment is often significant due to the vital structures involved, identification of a microbial pathway to disease may offer new insights into targeted therapies and primary prevention.

Prior work investigating the microbiome of head and neck cancer, including the largest cohort that was previously reported by our group, provided descriptive evidence of the “in”vironment of the head and neck at higher-order taxa and suggested that microbial variation correlates with clinical outcomes and gene methylation status [17]. Smaller studies that have used superficial sampling of oral cavity cancers by means of oral swabs observed differences in taxonomic abundance between normal and tumor surfaces primarily at the phylum level [18, 19]. However, bacteria in the head and neck are clearly not limited to the mucosal surface, but in fact populate deep tissue [17, 20, 21]. These findings are supported by our prior pilot study as well as a recent investigation composed of 29 patients with exclusively laryngeal cancer that demonstrated phylum and genus-level changes in tumor relative to normal tissue [22]. However, the significance of the findings from these studies is unclear. Furthermore, the microbiome content of cancerous mucosal tissue compared to adjacent histologically normal tissue has not been examined outside of the setting of laryngeal cancer.

With increasing evidence that a rich community of bacteria exists within head and neck tissues and may contribute to carcinogenesis, we now seek to identify microbiomic differences between tumor and histologically normal tissue in a large cohort of patients with HNSCC of the oral cavity, oropharynx, hypopharynx, and larynx. In this study, we report on the largest human tissue microbiome study in HNSCC patients, with 16S ribosomal DNA (rDNA) amplicon sequencing of paired normal-tumor tissue samples from 121 unrelated participnts. With these data, we correlate whole microbiome communities of head and neck tissue with clinical outcome measures of HNSCC, in order to test the hypothesis that microbiomes either alter or have been altered by both the presence and extent of HNSCC.

## Methods

### Patient cohort and sample collection

From 2003 to 2014, consecutive HNSCC patients were enrolled into a tissue biorepository collection. The tissue banking protocol was designed specifically to maintain sterility for downstream microbiome analysis. All tissues banked were required to be collected from the oral cavity, oropharynx, hypopharynx, or larynx. In this registry, 169 individuals had available paired adjacent normal and tumor tissue. A total of 30–50 mg each of paired tumor and normal tissue, approximately 2 cm from the tumor edge, were sterilely collected in the operating room, classified via pathology review, flash frozen, and stored at –80 °C. Relevant clinicopathologic features were collected prospectively at the time of diagnosis. Tumor node metastases staging was determined for each primary tumor based on American Joint Committee on Cancer guidelines [23]. Missing data were filled in via retrospective chart review; individuals without available data were noted as such in Table 1.

**Table 1:** Demographics and clinical characteristics of patients^a^

Variable	Included (n = 121)	Excluded (n = 38)	*p* value
Age (years)	63 ± 11	62 ± 13	0.73
Male	74 (64)	25 (74)	0.31
Race			0.07
White	71 (91)	19 (100)	
Black	7 (9)	0 (0)	
Localization			0.32
Oral cavity/Oropharynx	72 (65)	26 (74)	
Floor of mouth	5	2	
Tongue	42	11	
Tonsil	13	8	
Oral cavity NOS	12	5	
Hypopharynx/Larynx	38 (35)	9 (26)	
Hypopharynx	4	2	
Larynx	34	7	
T-stage			0.59
Low T-stage	44 (40)	15 (45)	
T0	4	2	
T1–T2	42	13	
High T-stage (T3–T4)	66 (60)	18 (55)	
N-stage			0.58
Node negative (N0)	56 (51)	15 (45)	
Node positive	54 (48)	18 (55)	
N1–N2	51	18	
N3	3	0	
Overall stage			0.16
I–II	24 (24)	4 (13)	
III–IV	78 (76)	28 (88)	
Previous treatment			
Operation	21 (19)	4 (12)	0.37
Chemotherapy	24 (21)	3 (9)	0.09
Radiotherapy	30 (26)	8 (24)	0.78
Smoking history			0.08
Current	18 (16)	1 (3)	
Past	68 (60)	24 (71)	
Never	29 (25)	9 (26)	
Alcohol use			0.96
Heavy	10 (9)	4 (12)	
Social	57 (50)	16 (47)	
History	10 (9)	3 (9)	
Never	37 (32)	11 (32)	

Values are presented as means ± standard deviations or number (percent)

^a^Data are missing for the following variables, indicated as “variable name: # missing in included group/# missing in excluded group”: Age: 7/5, Gender: 6/4, Localization: 11/3, Race: 43/19, T-stage 9/5, N-stage 11/5, Overall stage: 19/6, Prior operation: 8/5, Prior chemotherapy: 5/4, Prior radiation: 5/4, Smoking history: 6/4, Alcohol use: 7/4. Percentages are calculated from denominator of samples with known data

*NOS* not otherwise specified

### DNA extraction

Total DNA was extracted with modifications from a previously described protocol [17]. Bead homogenization of tissues was performed with a TissueLyser II (Qiagen, Venlo, The Netherlands). Also added was a yeast cell wall lysis step using the Masterpure Yeast DNA Purification kit (Epicentre, Madison, WI, USA) [24]. All beads, tubes, and non-enzymatic reagents were treated with ultraviolet light for at least 30 min prior to use [25]. Reagent controls were confirmed by 16S polymerase chain reaction (PCR) to be absent of contaminating bacteria.

### 16S rRNA gene sequencing

PCR of the V1–V4 hypervariable regions of the 16S rRNA gene was performed with previously published primers [17]. PCR was performed under the following conditions: 95 °C for 5 min, followed by 32 cycles of 95 °C for 1 min, 55 °C for 40 s, 70 °C for 80 s, and an extension of 72 °C for 10 min. PCR products were electrophoresed on a 1% agarose gel, purified using a Zymoclean DNA Gel Recovery kit (Zymo, Orange, CA, USA), and cloned into a StrataClone pSC vector (Agilent, Santa Clara, CA, USA) [17]. From an initial 169 pairs of patient samples, a total of 318 tissue samples from 159 distinct patients had positive 16S rDNA PCR product recovery. Ninety-five colonies were picked per tissue sample. Plasmid inserts were PCR amplified using standard T3/T7 primers, then Sanger sequenced (ABI3730xl, Life Technologies, Carlsbad, CA, USA).

### Bioinformatic analysis

Reads were filtered for quality, trimmed, and compiled using a custom python script. Depth of coverage was set at 60 sequences or higher based on leveling off of the Shannon diversity index at 60 reads. Due to this cutoff, a total of 242 tissue samples from 121 distinct patients were included in the final analysis. Subsampled open-reference operational taxonomic unit (OTU) picking [26] against Greengenes (version 13.8) [27, 28] at 97% similarity threshold using UCLUST [29], alignment with PyNAST [30], phylogenetic tree construction using FastTree (version 2.1.3) [31], and subsequent computation of alpha (Shannon diversity index, phylogenetic diversity) [32, 33] and beta diversity measures (weighted and unweighted Unifrac distances) [34, 35] was performed using QIIME (version 1.9.1) [36].

### Statistics

Student’s *t*-tests and likelihood ratio tests were used to compare continuous and categorical demographics/clinical factors, respectively, between patient samples included in the final analysis and those excluded due to insufficient reads. Student’s *t*-tests were used to compare Shannon index and phylogenetic diversity between tumor and non-tumor samples at a sequencing depth of 60 with ten iterations per sample. Distance matrices of the tumor and non-tumor samples were compared using the Adonis statistical method [37]. This method is similar to non-parametric analysis of variance (ANOVA) and relies on F-tests based on sequential sums of squares derived from 1000 permutations on the weighted and unweighted UniFrac distance matrices, with the null hypothesis that there is no difference in community structure between groups. To compare relative abundances of taxa between matched tumor-normal pairs, we used the non-parametric two-sided Wilcoxon signed-rank test. To compare relative abundances of taxa between samples of different T-stages, we used the Wilcoxon rank-sum.

All analyses were conducted in JMP Pro 12 (SAS Institute Inc., Cary, NC, USA) or R version 3.2.2. All statistical tests were two-sided, with a *p* value < 0.05 or false discovery rate (FDR) adjusted *q* < 0.05 considered statistically significant. All graphs were created using the R package lattice [38]. The cladogram was created using GraPhlAn on Galaxy [39, 40].

## Results

### HNSCC microbiomes are similar on a phylum-level to those in previous studies of human oral flora

**Fig. 1:**
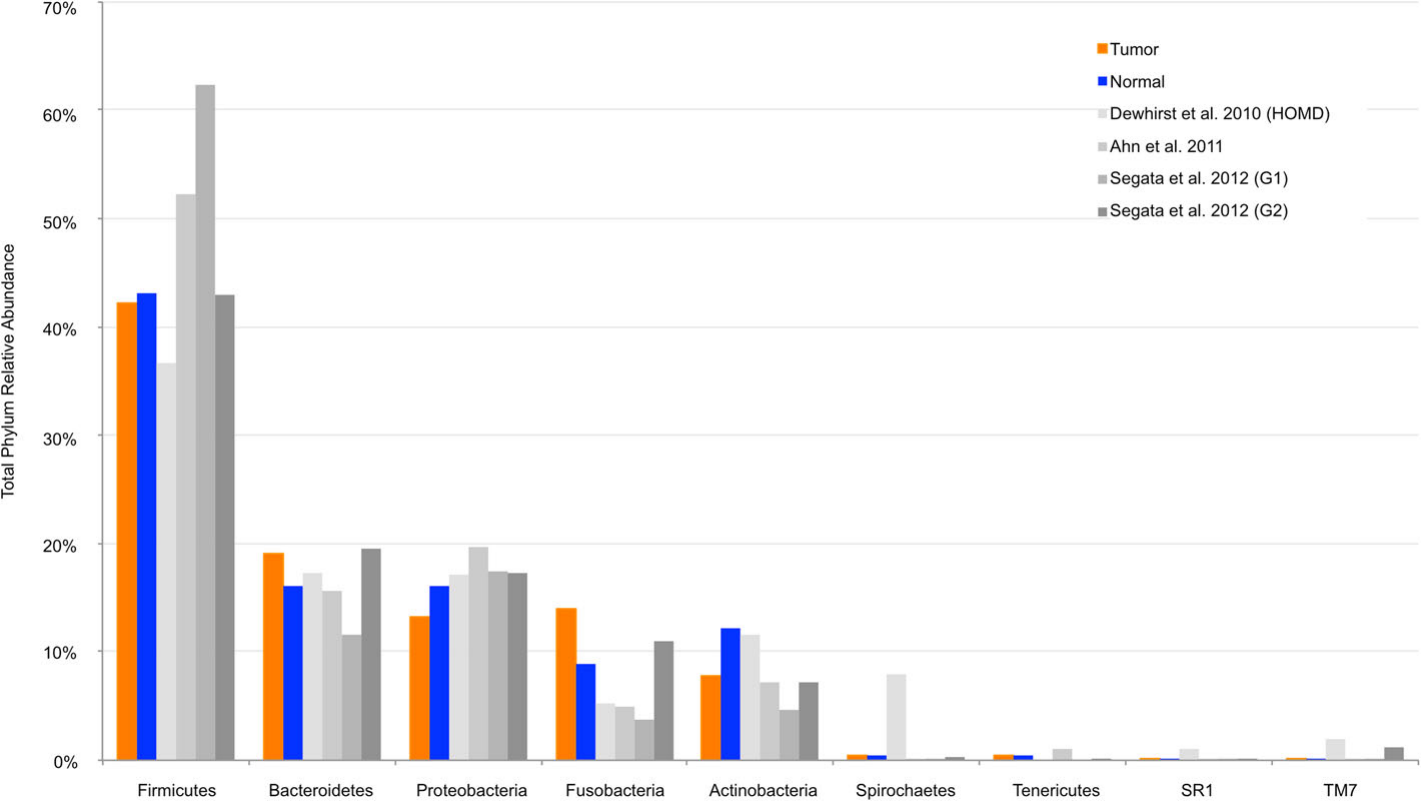
Relative abundances of major phyla in the human oral microbiome. *Bar plot* of relative abundances of major phyla in the oral microbiome observed in this study and three previously published series. There were similar relative abundances of the most common phyla among tumor (*orange*) and adjacent normal (*blue*) tissue from this study. Additionally, these abundances were similar to previously published series describing the oral microbiome

We analyzed sterilely collected, paired fresh-frozen normal-tumor samples from 121 patients with HNSCC. These patients were not significantly different on any demographic or clinical factors when compared to the 38 patients excluded based on low read count (Table 1). The taxonomic composition of our HNSCC samples is similar to that identified in our previous pilot study of HNSCC [17], as well as with data from previously published studies on the human oral microbiome [41–43]. Firmicutes is the predominant phylum, followed by Bacteroidetes and Proteobacteria, then by Fusobacteria and Actinobacteria, in both tumor and adjacent normal samples from HNSCC patients as well as in prior studies (Fig. 1). Phyla falling under 0.1% relative abundance in our dataset were not included in this analysis.

### HNSCC tumor and paired-normal tissue are not significantly different on measures of alpha or beta diversity

The average number of reads for the 242 patient samples in the final analysis was 83 ± 11 and did not differ between tumor (84 ± 13) and normal (83 ± 7) samples (*p* = 0.48). The average read length was 745 ± 117. To determine whether overall mean diversity was different in tumor and adjacent normal tissue of HNSCC patients, we compared two measures of alpha diversity: Shannon index (H) which measures the evenness and richness of a population; and phylogenetic diversity (PD) which takes the phylogenetic relationship between taxa into account. We found no significant difference in measures of alpha diversity between tumor (H = mean 3.72 ± standard error 0.78, PD = 6.42 ± 1.88) and normal (H = 3.87 ± 0.74, PD = 6.62 ± 1.96) tissue (H: *p* = 0.13, PD: *p* = 0.42).

**Fig. 2:**
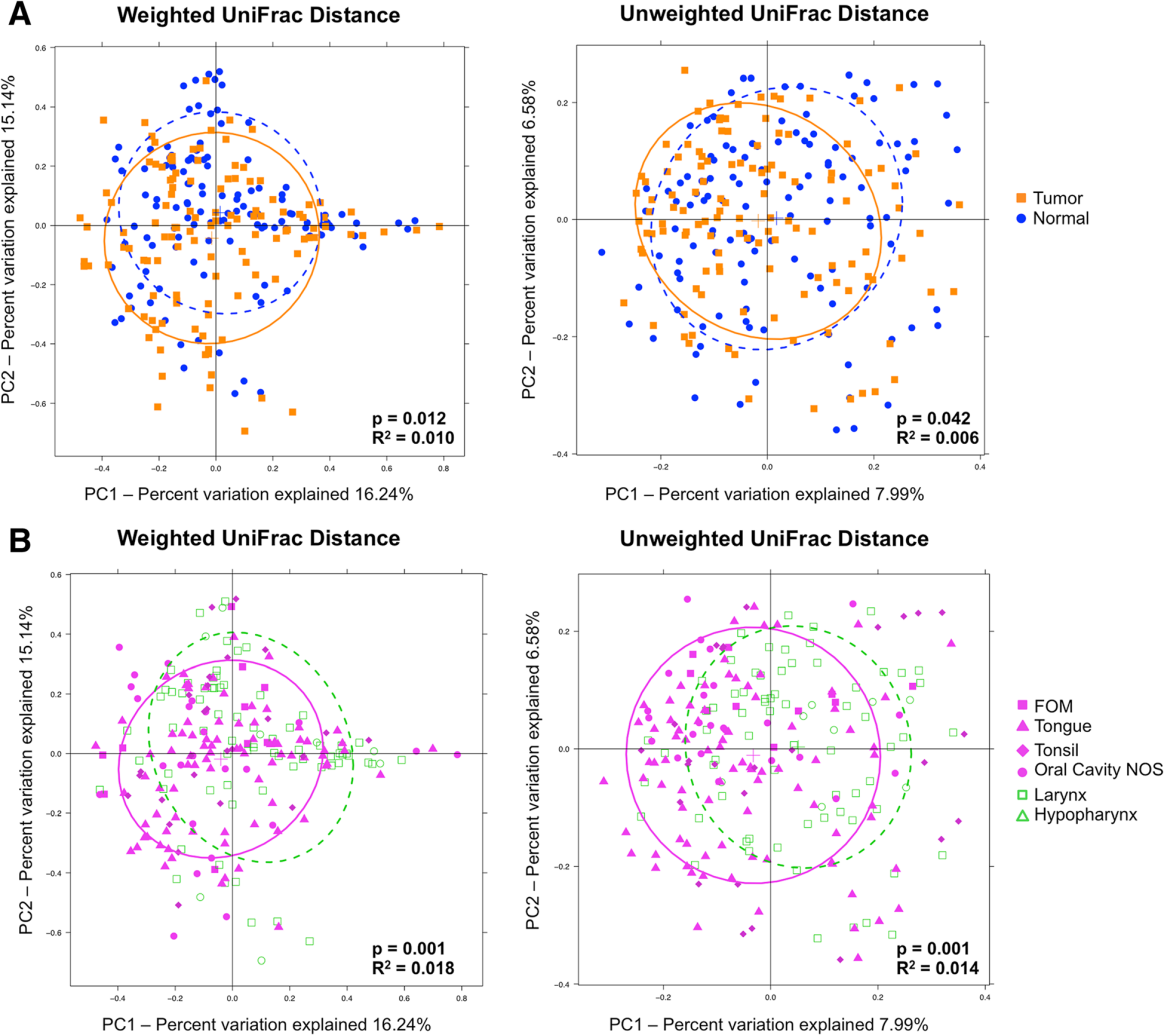
*PCoA plots* of weighted and unweighted UniFrac distances of tumor and normal samples. Overall oral microbiomic diversity of patient samples as represented by PCoA of weighted and unweighted UniFrac distances. In panel (**a**), each *point* represents a single tumor (*orange square*) or adjacent normal (*blue circle*) sample, with *plus sign* and *ellipses* (*orange solid line* = tumor, *blue dashed line* = normal) representing the fitted mean and 68% confidence interval of each group, respectively. Adonis testing revealed statistically significant clustering based on the tumor/normal grouping (weighted *p* = 0.012, unweighted *p* = 0.042), but this clustering only explained a small proportion of the overall variation among samples (weighted R^2^ = 0.010, unweighted R^2^ = 0.006). In panel (**b**), each *point* represents a single oral cavity/oropharyngeal (*magenta*) or hypopharyngeal/laryngeal (*green*) sample, with *plus sign* and *ellipses* (*magenta solid line* = oral cavity/oropharynx, *green dashed line* = hypopharynx/larynx) representing the fitted mean and 68% confidence interval of each group respectively. The different shapes provided by the legend delineate smaller sub-categories of each location. Adonis testing revealed statistically significant clustering of oral cavity/oropharyngeal samples relative to hypopharyngeal/laryngeal samples (weighted *p* = 0.001, unweighted *p* = 0.001), but this clustering only explained a small proportion of the overall variation among samples (weighted R^2^ = 0.018, unweighted R^2^ = 0.014)

To test whether overall bacterial taxa composition was different between tumor and normal tissue, we used principal coordinates analysis (PCoA) on weighted and unweighted Unifrac distances. We found that, although statistically significantly different on both weighted (*p* = 0.012) and unweighted (*p* = 0.042) measures, the tumor/normal grouping explained only a small proportion of the overall variation in the samples (Fig. 2a, Additional file 1: Figure S1A, B). This difference was also similarly significant (weighted *p* = 0.001, unweighted *p* = 0.001) but non-explanatory when comparing PCoAs of samples by whether they were from the oral cavity/oropharynx or the hypopharynx/larynx (Fig. 2b).

### Relative abundance of specific taxa differs between tumor and paired normal tissue

**Fig. 3:**
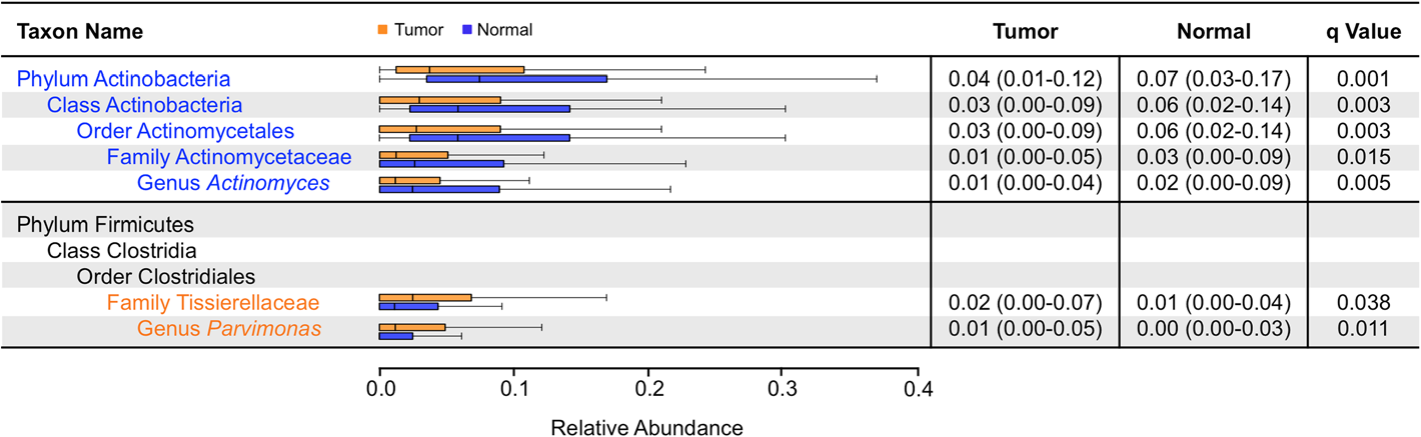
Significant taxa by Wilcoxon signed-rank in paired tumor and normal tissue. *Box plots* representing relative abundances of taxa observed to be significantly different between tumor (*orange*) and adjacent normal (*blue*) samples by paired Wilcoxon signed-rank testing after correction for FDR. *Dark vertical lines* represent the median, with the *box* representing the first (Q1) and third (Q3) quartiles, and the outer fences 1.5 × interquartile range. Outliers are not plotted. Values are reported as median (Q1–Q3), with q values representing significance of Wilcoxon signed-rank comparing tumor and normal relative abundances for each taxon after FDR correction. Taxa names are colored based on the group in which they are overrepresented

Next, we compared the relative abundances of 372 individual taxa between matched pairs of tumor and adjacent normal tissue, finding differences in ten genera, 12 families, eight orders, five classes, and three phyla by Wilcoxon signed-rank testing (Additional file 2: Figure S2). Only 2/10 genera were significant after adjusting for FDR: *Actinomyces* and *Parvimonas*. The genus *Actinomyces*, along with its parent family Actinomycetaceae, order Actinomycetales, class Actinobacteria, and phylum Actinobacteria, was depleted in tumor compared to matched normal tissue. In contrast, the genus *Parvimonas*, along with its parent family Tissierellaceae, was increased in tumor compared to normal tissue (Fig. 3).

**Fig. 4:**
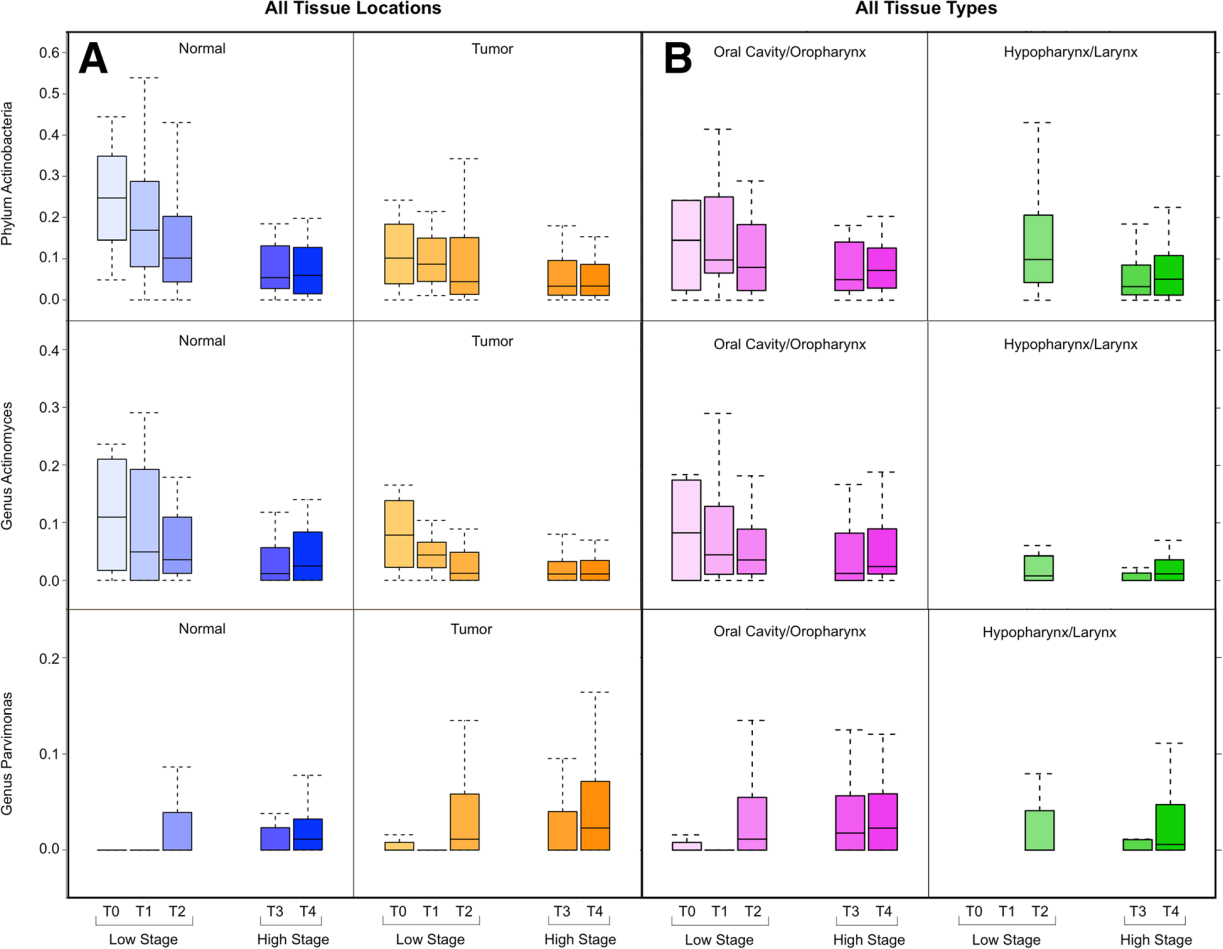
Relative abundances of differentially represented taxa stratified by T-stage. *Box plots* representing relative abundances of phylum Actinobacteria (*top*), genus *Actinomyces* (*middle*), and genus *Parvimonas* (*bottom*) stratified by T-stage. *Dark horizontal lines* represent the median, with the *box* representing the first (Q1) and third (Q3) quartiles, and the outer fences 1.5 × interquartile range. Outliers are not plotted. **a** Samples stratified by type (normal = *blue*, tumor = *orange*), with *darker colors* representing higher T-stage. In both normal and tumor samples, the relative abundances of Actinobacteria and *Actinomyces* decrease, while *Parvimonas* increases, with increasing T-stage. **b** Samples stratified by location (oral cavity/oropharynx = *magenta*, hypopharynx/larynx = *green*), with *darker colors* representing higher T-stage. In the oral cavity/oropharynx, the relative abundances of Actinobacteria and *Actinomyces* decrease, while *Parvimonas* increases, with increasing T-stage. In hypopharyngeal/laryngeal samples, only Actinobacteria is decreased with increasing T-stage

After identifying taxa that were significantly different between tumor and paired normal tissues, we performed a stratified analysis to investigate the relationship between tumor stage and the relative abundances of these taxa. We observed that samples from low-stage (T0–2) patients had significantly increased relative abundance of the genus *Actinomyces* compared to samples from high-stage (T3–4) patients (median 3.3% versus 1.2%, *p* = 0.005). The parent taxa of the genus *Actinomyces* were also significantly relatively increased in low-stage patients compared to higher stages, up to the phylum level. In contrast, the genus *Parvimonas* was significantly relatively decreased in samples from low-stage patients compared to high-stage patients (median 0.0% versus 1.1%, *p* = 0.023). The relationship between these taxa and T-stage remained consistent when stratifying by tumor versus paired-normal tissue (Fig. 4a). This difference was statistically significant in the normal group (phylum Actinobacteria *p* = 0.002, genus *Actinomyces p* = 0.023, genus *Parvimonas p* = 0.033), but only approached significance in the tumor group (phylum Actinobacteria *p* = 0.067, genus *Actinomyces p* = 0.052, genus *Parvimonas p* = 0.247).

As T-stage was significantly associated with tissue location (oral cavity/oropharynx versus hypopharynx/larynx), we proceeded to stratify samples based on tissue location (Fig. 4b). We observed that relative abundances of the phylum Actinobacteria, genus *Actinomyces*, and genus *Parvimonas* were consistently lower at hypopharyngeal/laryngeal locations relative to the oral cavity/oropharynx. However, when analyzing oral cavity/oropharynx samples alone, Actinobacteria and *Actinomyces* approached significance in low-stage patients relative to high-stage patients (*p* = 0.100, *p* = 0.192) and *Parvimonas* remained significantly relatively decreased among low-stage patients compared to high-stage patients (*p* = 0.006). When analyzing hypopharyngeal/laryngeal samples alone, Actinobacteria remained significantly relatively increased in low-stage patients (*p* = 0.031), while *Actinomyces* and *Parvimonas* were not significantly different between low-stage and high-stage groups (*p* = 0.645, *p* = 0.790).

## Discussion

In this study, we sought to describe the oral microbiome of individuals with HNSCC and to compare the local microbiome of their tumors with neighboring normal tissue. We hypothesized that tumor tissue would have a microbiome unique from that of adjacent normal tissue and be more pronounced in higher stage disease. The simple comparison of tumor versus adjacent normal tissue did not reveal major shifts in overall diversity (Shannon index or phylogenetic diversity) or in microbiomic content. However, matched pairs analysis of individual tumor-normal pairs revealed significant differences in relative abundance of specific taxa, namely the genera *Actinomyces* and *Parvimonas*. These differences were more pronounced in patients with a higher T-stage.

The phylum-level oral microbiome of individuals in our study was similar to those reported previously. Dewhirst et al. reported on the Human Oral Microbiome Database, which consisted of 633 Sanger-sequenced oral 16 s rRNA gene libraries from various head and neck sites of patients of various states of health and disease [41]. Ahn et al. analyzed oral washes from 20 individuals (ten with malignant or premalignant oral lesions, ten healthy controls) using both 16 s rRNA pyrosequencing and a custom DNA microarray [42]. Segata et al. found in their study of over 200 healthy adults that the adult digestive tract microbiome differed according to location of sampling; group 1 (G1) sites (buccal mucosa, keratinized gingiva, and hard palate) had increased relative abundance of Firmicutes and decreased relative abundance of other phyla as compared to group 2 (G2) sites (saliva, tongue, tonsils, and throat) [43]. The phyla-level composition of our study population was most similar to Segata et al.’s G2 series, despite the fact that they used next-generation sequencing (NGS) instead of Sanger sequencing, used swabs instead of surgically excised tissue, and had healthy controls instead of patients with HNSCC. This was not surprising given that the majority of our patient tissues were from tongue and tonsil locations (Table 1), as in Segata et al.’s G2 series [43].

We did not observe any differences in overall diversity of tumor and adjacent normal tissue samples from HNSCC patients. On analysis of alpha diversity measures, we found that the Shannon diversity index of our samples was similar to previously reported measures [44, 45]. Median phylogenetic diversity of our samples was lower than the median described by Takeshita et al. in their study of over 2000 healthy Japanese individuals [46]. This suggests that our study, which uses Sanger sequencing and thus has fewer reads, may under-predict the true phylogenetic diversity of patient samples. However, this difference may also be due to differences in patient disease status, ethnicity, diet, and/or sample type (saliva versus tissue).

Although our samples did cluster into statistically significant normal and tumor groups based on weighted and unweighted UniFrac distances, this grouping only explained a small proportion of the overall variation seen in our samples (Fig. 2). This is unsurprising considering the relative proximity (2 cm) of these two categories of tissue. In fact, the relative histological similarity of adjacent “normal” tissue to neighboring tumor tissue was first described in oral epithelia, and given the name “field cancerization” [47]. Coined to designate large areas of premalignant tissue with altered histology adjacent to malignant tumor tissue, field cancerization may also apply to the resident microbiome. These data imply that more similarities than differences exist between the overall oral microbiomes of tumor and adjacent normal tissues from the same patient, consistent with what has been described previously in a smaller series [18].

Despite similarities on the community level, we observed differences between matched pairs of tumor and normal samples on the individual taxon level. Relative abundances of the genus *Actinomyces*, along with its parent taxa up to the phylum level, were significantly decreased in tumor as compared with normal samples (Fig. 4). Schmidt et al. also described a decrease in the relative abundance of 11 OTUs from the phylum Actinobacteria in swabs of tumor sites as compared to contralateral normal mucosa in 13 individuals with HNSCC [18]. Similarly, Gong et al. observed decreased levels of Actinobacteria in 27 patients with laryngeal carcinoma compared to 28 participants with vocal cord polyps [19].

Members of the genus *Actinomyces* are human commensals in the oropharynx, gastrointestinal, and female genital tracts, but can rarely cause subacute to chronic infections in the setting of mucosal disruption [48]. While neither this investigation nor the abovementioned studies can establish the nature or timeline of the relationship between depletion of *Actinomyces* and malignancy, it is possible that *Actinomyces* spp. exert a protective effect through the secretion protease-inhibitors that inhibit tumorigenesis [49]. Alternatively, *Actinomyces* spp. could be out-competed by faster-growing oral commensals at the relatively acidic, hypoxic, and glucose-starved tumor microenvironment [50]. This hypothesis would be most consistent with our observation that relative abundances of *Actinomyces* and Actinobacteria were not only decreased in tumor compared to adjacent normal tissue, but more so in higher T-stage samples (Fig. 4, top and middle). The effect of T-stage was more pronounced in adjacent normal tissue than in tumor, suggesting that *Actinomyces* depletion may precede tumor invasion. Importantly, node positivity was not associated with *Actinomyces* relative abundance, indicating that it may not have a role in the tumor’s metastatic potential.

Although a statistically significant difference in the relative abundance of genus *Parvimonas* was observed between tumor and adjacent normal tissue, the absolute difference was small and may not be clinically relevant. In addition, although the increase in genus *Fusobacterium* and its parent taxa up to the phylum level in tumor samples was not statistically significant after correction for multiple comparisons, this finding is consistent with previous reports [18] and may be important in the context of what is known about *Fusobacterium* and colorectal cancer [2, 3, 5].

This investigation represents the largest study of the microbiome of patients with HSNCC to date, with 121 matched tumor and adjacent normal samples. Moreover, a non-parametric matched pairs analysis was conducted, in contrast to prior studies, which allowed us to control for demographics, clinical characteristics, lifestyle factors, and inherent inter-individual microbiomic variability when comparing tumor and normal samples. In contrast to prior investigations that used swabs or oral rinses, we used surgically excised, histologically verified, tumor and adjacent normal tissue. This offers the capacity to directly sample the tumor microenvironment and compare it to the microenvironment of adjacent normal tissue.

At the time of protocol initiation, NGS was less widely available relative to Sanger sequencing. While this is a limitation of our investigation due to the relatively low read counts in our study, there is evidence to suggest that useful comparisons can be made at this sequencing depth [51]. Prior studies have demonstrated that low numbers of reads can accurately characterize communities at the phylum level and be used to uncover large-scale differences between communities through analysis of beta-diversity metrics [34, 52, 53]. Other studies have demonstrated that while Sanger sequencing (at a depth of 50 reads per sample) will miss rare species, it can capture most of the microbial diversity and accurately characterize abundances of predominant taxa [54, 55].

We acknowledge that the power to detect statistically significant differences in relative abundances is limited by the low read counts offered by Sanger sequencing. As such, the likelihood of false negatives in this study is quite high. However, despite low read counts, significant differences were observed, some consistent up to the phylum level, reflecting the large effect size of these differences.

## Conclusions

We conclude that the microbiomes of HNSCC tumor microenvironments are largely similar in overall diversity and bacterial composition to that of histologically normal adjacent tissue. However, we detected decreases in the genus *Actinomyces* and its parent taxa up to the phylum level and found that this decrease was more pronounced in higher T-stage samples. Further investigation is needed to validate these findings in a large series using NGS methods and to determine the biological relevance of this observed difference.

## Additional files

Additional file 1: Figure S1. Overall oral microbiomic diversity of patient samples as represented by PCoA of (**A**) weighted and (**B**) unweighted UniFrac distances. Each point represents a single tumor (*orange*) or normal (*blue*) sample, with connecting lines delineating a tumor/normal pair from the same patient. (TIFF 1177 kb)
Additional file 2: Figure S2. Cladogram depicting phylogenetic relationship of taxa identified as significantly different (*p* < 0.05) by Wilcoxon signed-rank testing in tumor relative to adjacent histologically normal tissue prior to correction for FDR. Each *concentric ring* of nodes represents a taxonomic rank, starting with kingdom at the very *center*. Moving outwards, the rings represent phylum, class, order, family, and genus. Nodes highlighted in *orange* are increased in tumor relative to normal samples. Nodes highlighted in *blue* are increased in normal relative to tumor samples. (TIFF 2213 kb)

## Abbreviations

H: Shannon index
HNSCC: Head and neck squamous cell carcinoma
PD: Phylogenetic diversity
