# Supplementary figures and images for: Microbiomic differences in tumor and paired-normal tissue in head and neck squamous cell carcinomas
Source: Genome Med. 2017 Feb 7;9:14. doi: 10.1186/s13073-017-0405-5 (PMC5297129; doi:10.1186/s13073-017-0405-5)

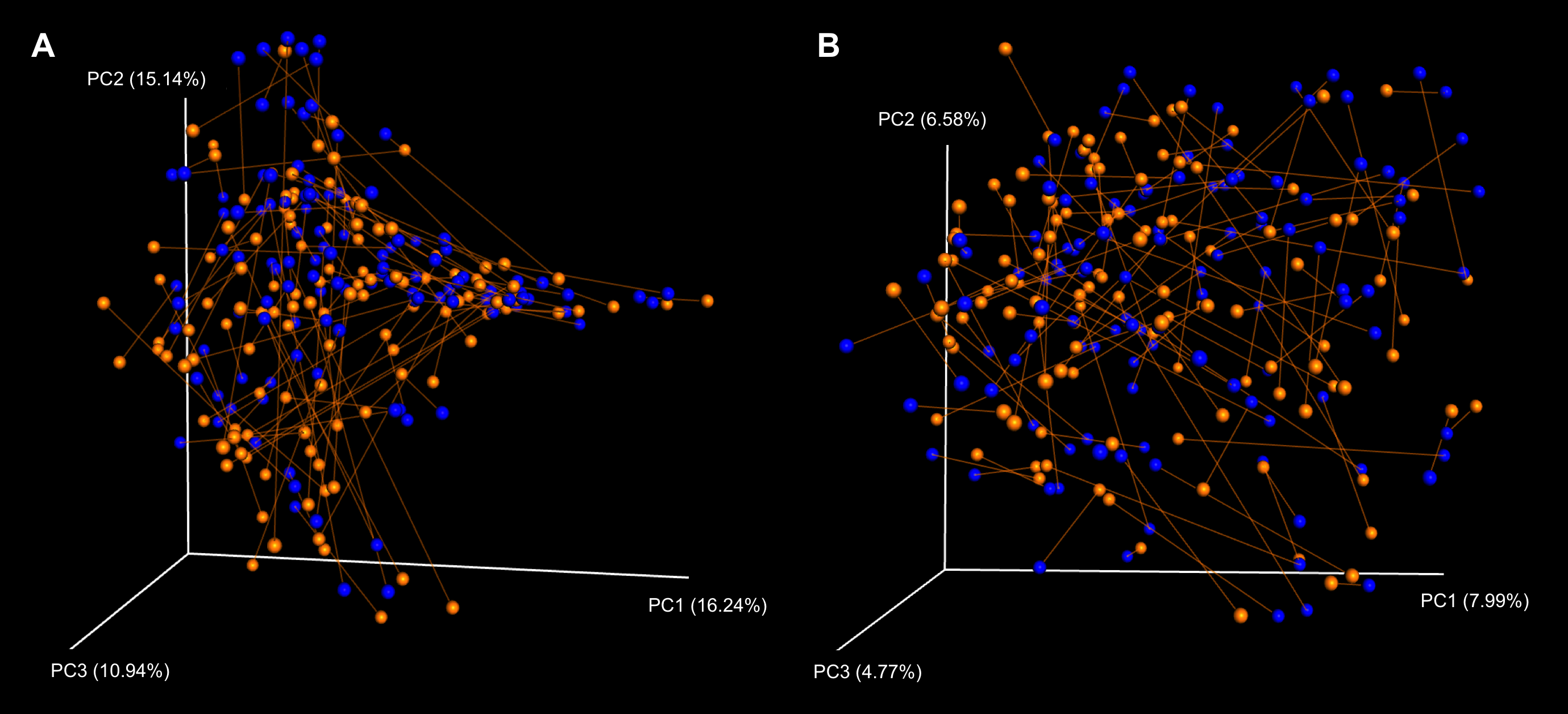

Supplement: Additional file 1: Figure S1. — Overall oral microbiomic diversity of patient samples as represented by PCoA of (A) weighted and (B) unweighted UniFrac distances. Each point represents a single tumor (orange) or normal (blue) sample, with connecting lines delineating a tumor/normal pair from the same patient. (TIFF 1177 kb) [file 13073_2017_405_MOESM1_ESM.tiff]

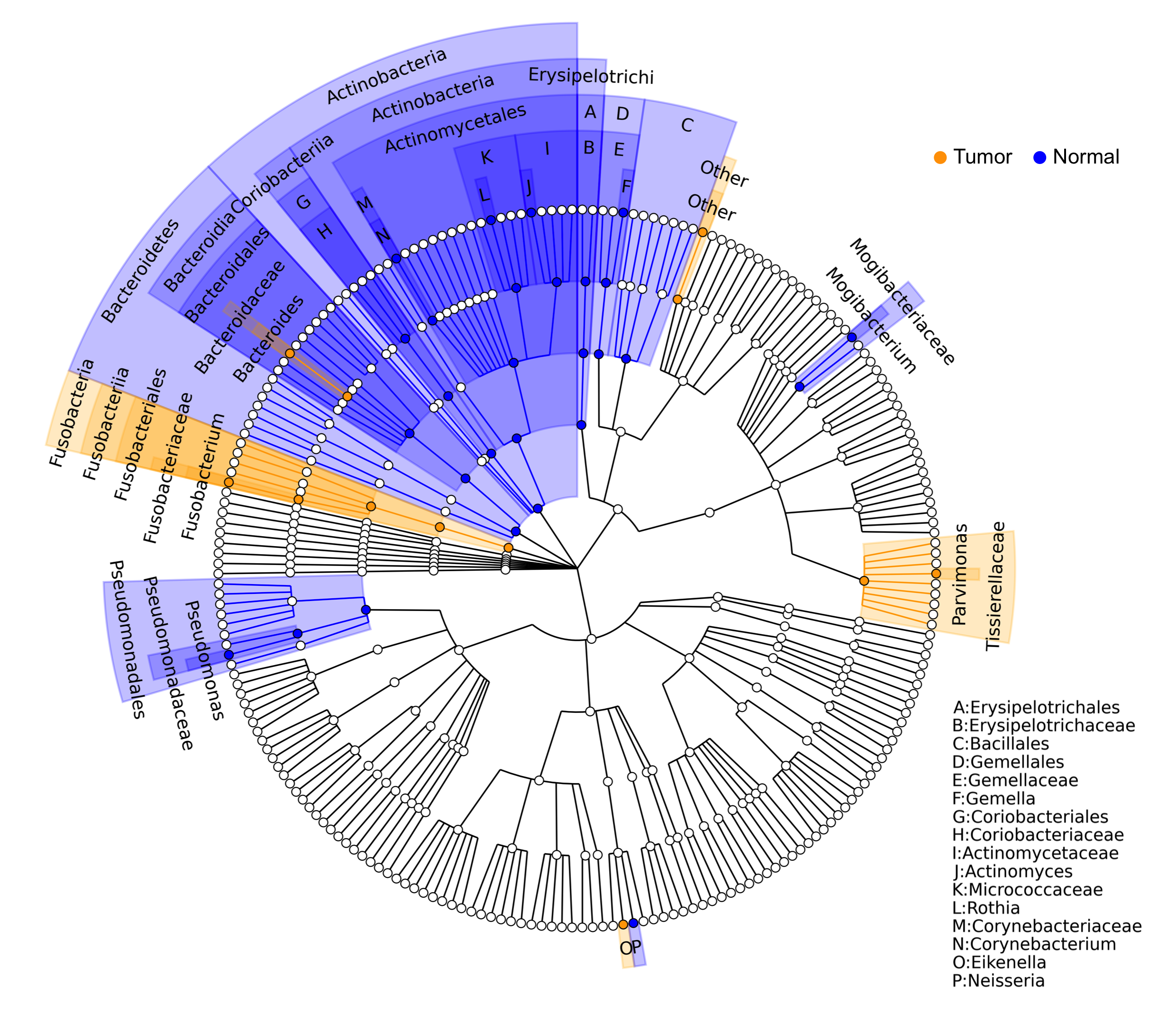

Supplement: Additional file 2: Figure S2. — Cladogram depicting phylogenetic relationship of taxa identified as significantly different (p < 0.05) by Wilcoxon signed-rank testing in tumor relative to adjacent histologically normal tissue prior to correction for FDR. Each concentric ring of nodes represents a taxonomic rank, starting with kingdom at the very center. Moving outwards, the rings represent phylum, class, order, family, and genus. Nodes highlighted in orange are increased in tumor relative to normal samples. Nodes highlighted in blue are increased in normal relative to tumor samples. (TIFF 2213 kb) [file 13073_2017_405_MOESM2_ESM.tiff]
